# Transsphenoidal meningoencephalocele protruding into the nasal cavity

**DOI:** 10.1259/bjrcr.20160082

**Published:** 2016-11-10

**Authors:** Vera Cruz e Silva, Ana Luís, Rita Mora Féria, Luís Marques, Martinha Chorão, Carla Reizinho, Joana Graça

**Affiliations:** ^1^Department of Neuroradiology, Egas Moniz Hospital, Lisbon, Portugal; ^2^Department of Neurosurgery, Egas Moniz Hospital, Lisbon, Portugal; ^3^Department of Pathology, Egas Moniz Hospital, Lisbon, Portugal

## Abstract

Spontaneous transsphenoidal meningoencephalcele is a rare entity, even rarer through the Sternberg’s canal, a congenital defect on the lateral wall of the sphenoid sinus. We report such a case in an obese 52-year-old female with spontaneous cerebrospinal fluid (CSF) rhinorrhoea and recurrent meningitis. Brain CT, MRI and CT cisternography were performed. Surgical correction and short-term follow-up were recorded. CT scan showed a defect on the lateral wall of the right sphenoid sinus filled with a soft tissue mass extending to the nasal cavity. MRI scan revealed brain parenchyma from the right temporal lobe herniated through the sphenoid bone defect. CT cisternography showed 270 mmH_2_O opening pressure and confirmed the CSF leakage. Surgical correction was performed with resolution of the symptoms. MRI and CT are complementary modalities for evaluating this entity, the first being the method of choice for meningoencephalcele diagnosis although bone defects are best depicted on CT scan. CT cisternography identifies the specific site of leak and confirms benign intracranial hypertension, consistently reported in meningoencephaloceles. Obesity and benign intracranial hypertension have been reported as a combined mechanism allegedly contributing to meningoencephaloceles through congenital skull base defects, by increasing intraabdominal pressure thus decreasing venous return, with augmented intracranial pressure and subsequent reduced absorption of the CSF.

## Clinical presentation

A 52-year-old female presented with an 8-year history of rhinorrhoea clinically consistent with cerebrospinal fluid (CSF) leakage and two episodes of meningitis. She also complained of mild headache worsening with flexion posture of the head, dizziness and, since last occurrance of meningitis, anosmia. No previous mild to severe head trauma or surgeries were reported. Other clinically relevant data were morbid obesity (body mass index 54 kg m^−^^2^), non-treated hypertension and early menopause (at 36 years). A positive glucose test confirmed CSF rhinorrhoea.

## Imaging findings

**Figure 1.**
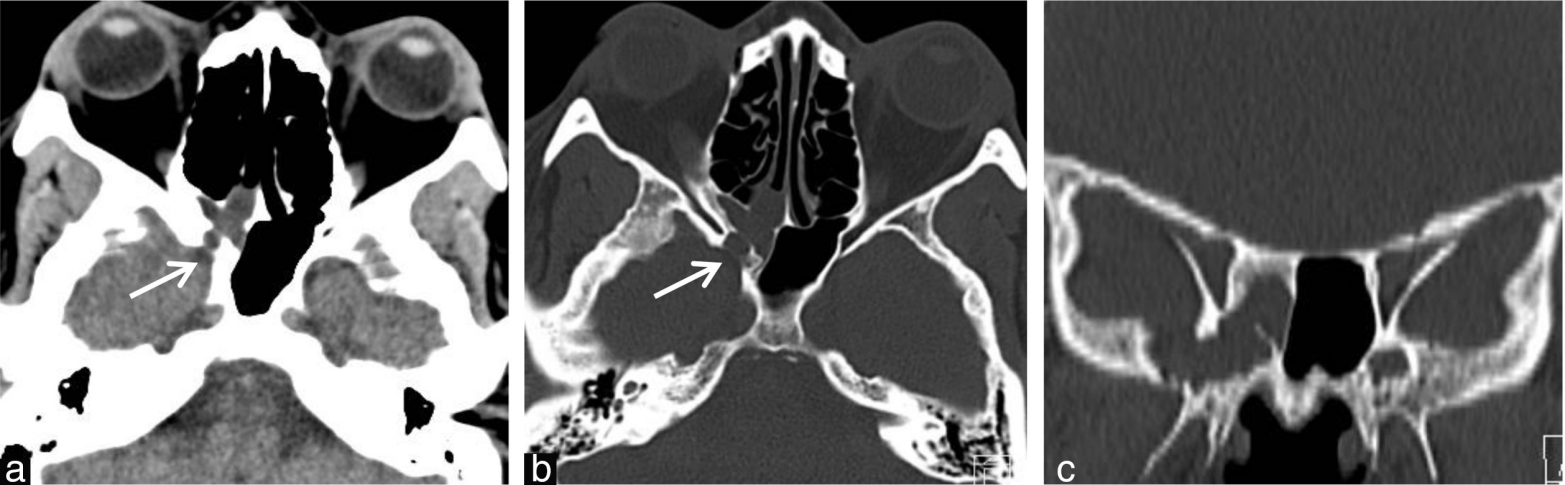
CT imaging of soft tissue (a) and bone windowing (b and c) showing a hypodense mass protrusion into the sphenoid sinus (arrows) through a skull base defect in its inferior and lateral wall—Sternberg’s canal.

**Figure 2.**
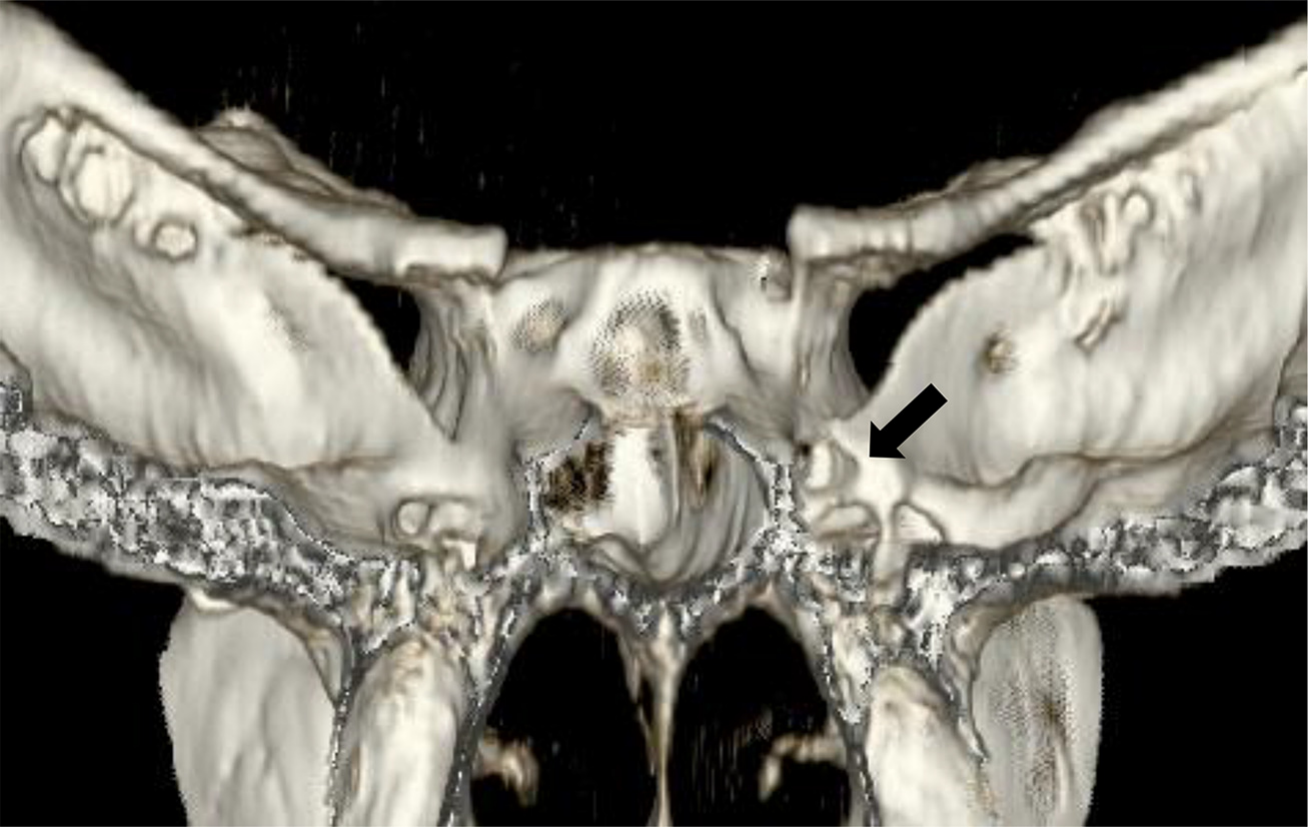
3D bone reconstruction of the skull base, posterior view. Arrow displaying the abnormal sphenoid bone defect on the right side, upper and medial to the foramen rotundum.

Head CT scan (SIEMENS AG, Munich, Germany) showed a sphenoid sinus bony defect on its posterior and lateral aspects, medial to foramen rotundum, through which a soft tissue density mass could be observed, connecting the temporal fossa with the right sphenoid sinus and nasal cavity, protruding into its roof (Figures 1 and 2). In addition, subtle focal areas of bone erosion adjacent to the defect were observed and interpreted as arachnoid granulations.

**Figure 3.**
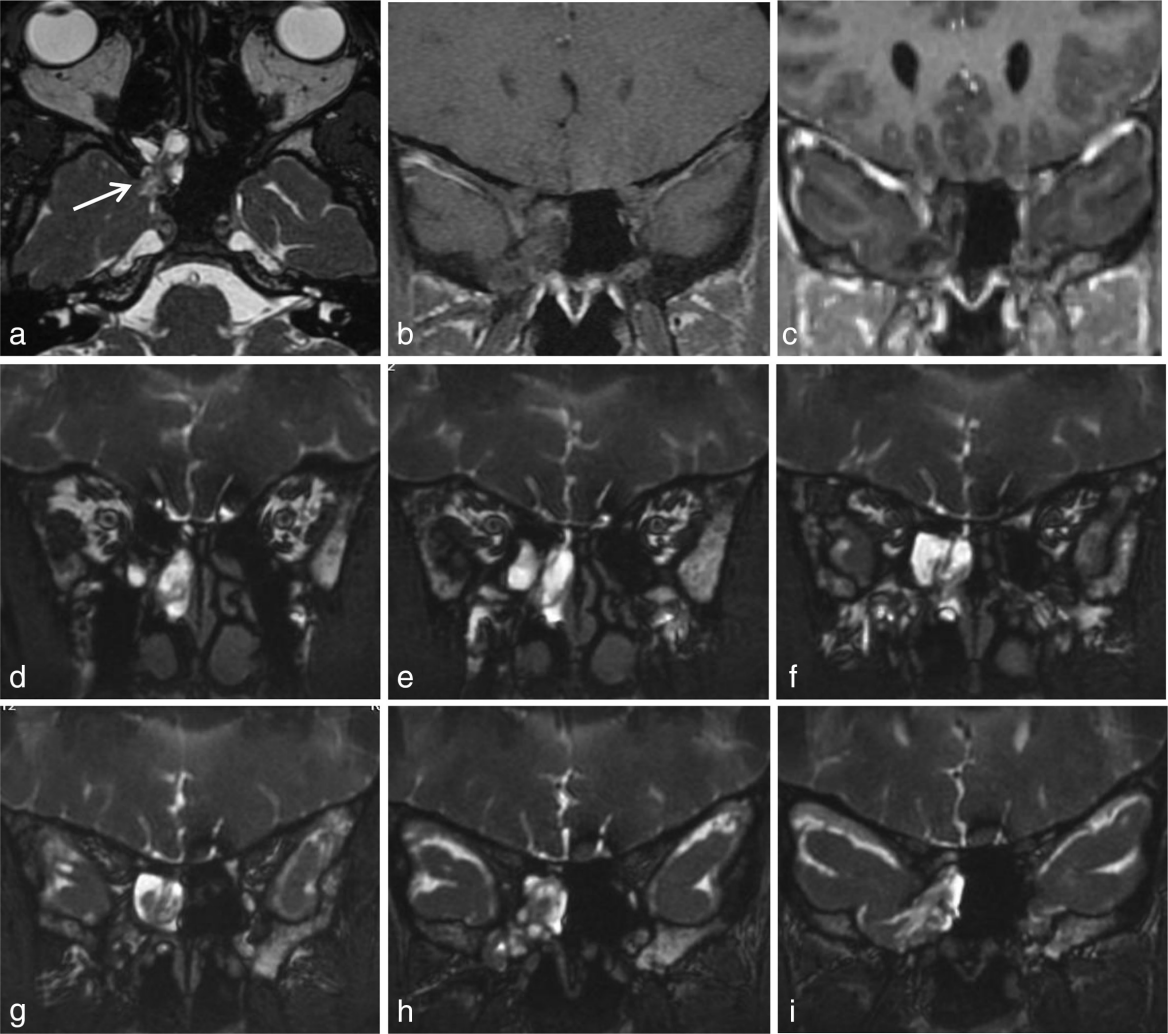
MRIof axial T_2_ weighted 3D high-resolution Fast Imaging Employing Steady-state Acquisition (FIESTA) (a), coronal *T*_1_ Fast Spin-Echo (FSE) before (b) and T1 3D Fast Spoiled Gradient-Recalled-Echo (FSPGR) after (c) gadolinium intravenous administration; reformatted coronal *T*_2_ weighted images FIESTA from the front to the back (d-i). Brain parenchyma herniation (arrow, a), without contrast enhancement, filling the sphenoid sinus and protruding in the nasal fossa through the enlarged sphenoidal ostium.

**Figure 4.**
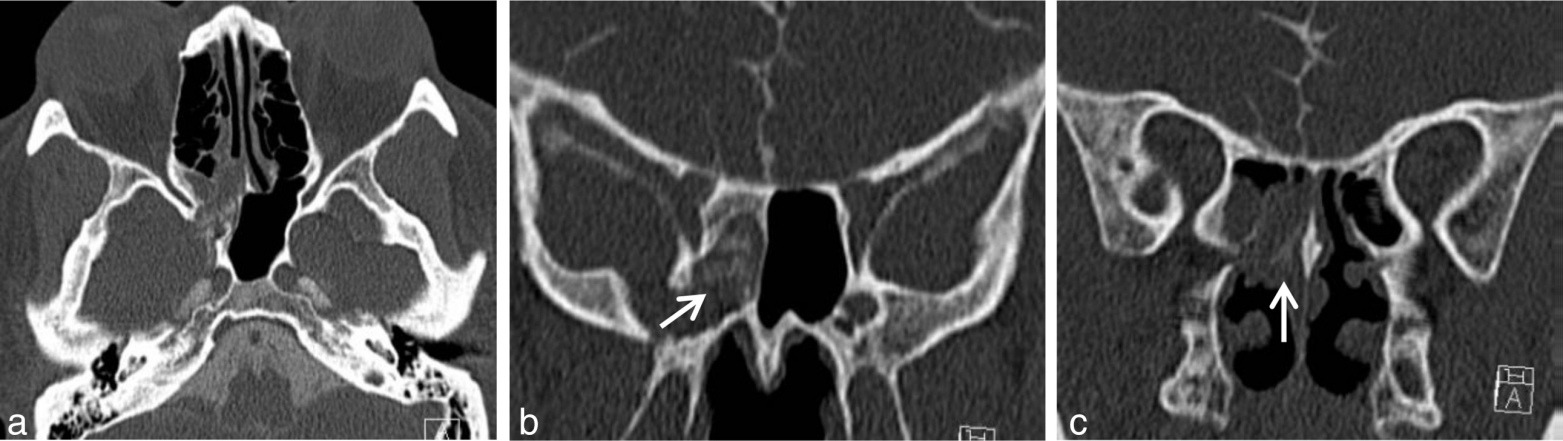
CT cisternography shows the axial (a) and coronal (b and c) views of non-ionic contrast in the sphenoid sinus (arrow, b) and nasal cavity (arrow, c) confirming cerebrospinal fluid leakage through the same bone defect.

MRI study performed (General Electric, Boston, Ma, USA) showed gliotic temporal brain parenchyma and a surrounding meningeal sac, without contrast enhancement, herniating through the previously identified sphenoid bone defect, with extension to the nasal cavity through the sphenoid ostium, consistent with meningoencephalocele of true transsphenoidal type (Figure 3). Additionally, a partially empty pituitary sella was detected. CT cisternography (Figure 4) demonstrated non-ionic contrast through the same bone defect into the sphenoid sinus and then to the nasal cavity, excluding other sites of CSF leakage. Opening pressure was measured and found to be elevated (270 mmH_2_O).

## Treatment and outcome

**Figure 5.**
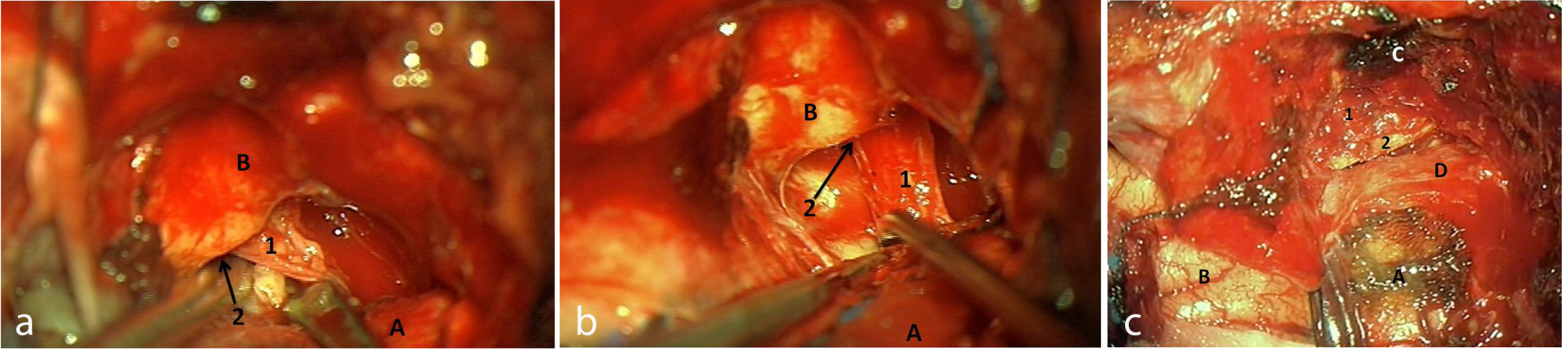
Surgical procedure with right pterional incision and temporal extension. (a) Extradural exposure of meningoencephalocele (1) through the dural defect and the persistent Sternberg’s canal (2) to the lateral recess of sphenoid sinus. A, temporal lobe; B, temporal fossa. (b) Extradural exposure of superior maxillary nerve (1) immediately before crossing foramen ovale (2), after resection of the meningoencephalocele. A, temporal lobe; B, temporal fossa. (c) Surgical repair of the bone defect after meningoencephalocele excision—temporal muscle flap, biological glue and temporal bone fragment (1)—and of the dural defect with artificial dura and biological glue. A, temporal lobe; B, frontal lobe; C, temporal fossa; D, temporal dura-mater.

**Figure 6.**
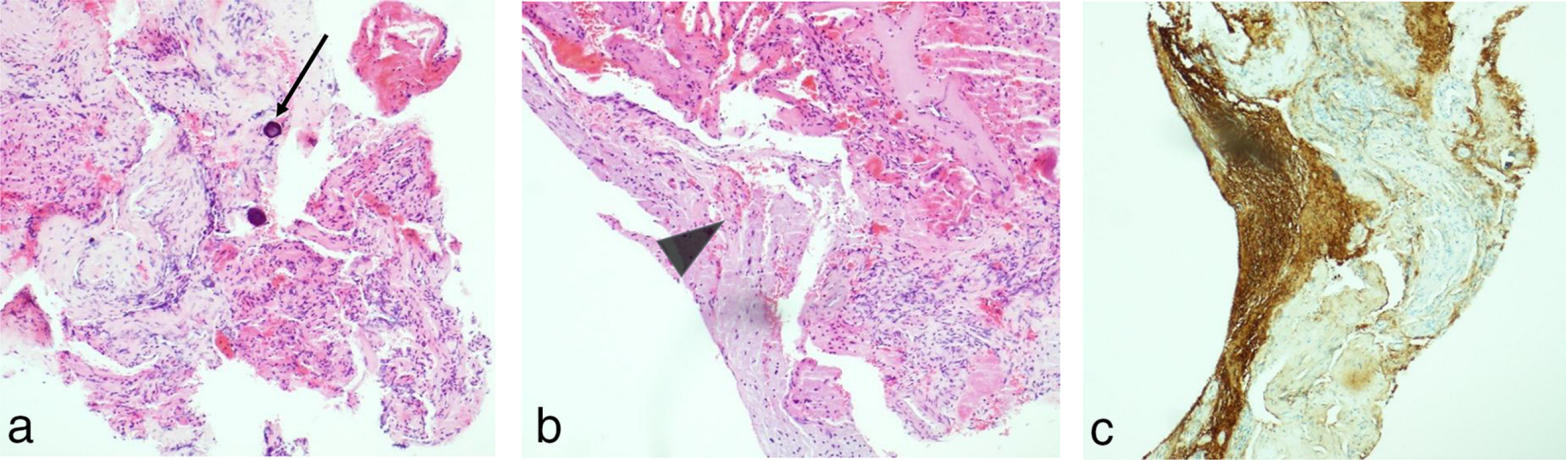
Histopathological examination of the surgically extracted tissue shows (a) meningeal tissue with psammoma bodies (arrow) and (b) brain tissue (arrow head) that was positive for glial fibrillary acidic protein (c).

The defect was corrected surgically (Figure 5), and the tissue examined histopathologically confirmed the meningoencephalocele (Figure 6). A short-term (22 months) follow-up confirms resolution of the symptoms as well as the efficacy of the surgical procedure.

## Discussion

Meningoencephaloceles consist of cerebral parenchyma and meninges herniating into the extracranial compartment through a cranial defect. They are divided into three main groups according to the defect location: basal, sincipital or occipital,^1^ the last being the most common.^2^ The basal type, accounting for 10% of all meningoencephaloceles,^3^ includes transethmoidals, sphenoethmoidals, frontosphenoidals and finally those involving exclusively the sphenoid sinus—transsphenoidals, intrasphenoidals, sphenoidal lateral and transalar. Depending on whether the meningoencephalocele is contained by the sinus or surpasses its wall protruding into the nasal cavity it is classified as intrasphenoidal or transsphenoidal, respectively.^4^ They may be congenital or acquired,^5^ the latter being more common and often due to traumatic skull base fracture, rarer after a neurosurgical intervention. Congenital meningoencephaloceles may occur isolated or in association with other congenital defects when an error occurs early during neural tube formation, most commonly associated to frontoethmoidal type, accompanied by facial malformation, hypertelorism and nasal obstruction.^6,7^ When no underlying cause is identified they are referred to as spontaneous meningoencephaloceles.

The sphenoid sinus is rarely a site of meningoencephaloceles and even rarer through the Sternberg’s canal, a congenital anomaly also called lateral craniopharyngeal canal, first described by Maximilian Sternberg in 1988^8^ as the result of incomplete fusion of greater wings of the sphenoid bone with basisphenoid by the 50th gestational day.^9^ In adults, it can be patent or vestigial up to 4% and 30%, respectively.^10^ By definition and according to literature two locations are hypothesized, either medial to foramen rotundum, defended by Barañano in its thousand patients series,^10^ or lateral to it, as stated by Tomazic and Stammberger.^11^ To our knowledge to date only 36 cases of meningoencephaloceles through the Sternberg’s canal were reported,^1,3,5,6,11–14^ but fewer of true transsphenoidal type with nasal cavity extension. Some involving the sphenoid sinus have been related to laterally extensive pneumatization in large sinuses; therefore contact between the Sternberg’s canal and the lateral recess of the sphenoid sinus can occur.^15^

The most common clinical presentation of meningoencephaloceles includes CSF rhinorrhoea; however, this entity is sometimes misdiagnosed and meningitis can be the first alarming clinical sign. When recurrent meningitis occurs it is highly suspicious of a cranial defect.^16^

As in our case, middle-aged obese (body mass index >30 kg m^−^^2^) females are consistent among previously published cases, many of them presenting with benign increased intracranial pressure and empty sella, as well as focal arachnoid granulations along the defect. An explanation could be the augmented intraabdominal and thoracic pressures leading to benign intracranial hypertension, obesity being the main risk factor.^11,17^ Another theoretical mechanism emphasizes the adipose tissue hormonal production in women, with relative hypoadrenalism inducing altered resistance to CSF dynamics and higher pressures at arachnoid villi, thus causing focal meningeal herniation.^10^ These mechanisms suggest the persistent benign intracranial hypertension upon an already weak structure of the cranium to be a relevant prognostic factor. Also, the altered CSF dynamics seems to be associated with empty or partially empty sella.^5^

CT can give great bony detail, defining the location and wideness of the defect; additional soluble contrast medium injection into the basal cisterns - cisternography—may confirm the CSF fistula, increasing the sensitivity to detect active leaks up to 85%.^18^ MRI, especially thin 3D *T*_2_ weighted images acquisition, allows further diagnosis concerning the soft tissue mass, whether it may contain brain parenchyma or only a meningeal sac.

Treatment is solely surgical and mandatory in order to prevent further central nervous system infection, which carries a reported 40% long-term risk of meningitis.^5^ Although endoscopic repair is sometimes preferred, transcranial approach allows direct access and improved visualization in cases presenting with extensive herniation and a wide defect.^3,9^ Surgical treatment was performed through transcranial approach (Figure 5a,b) and consisted of tissue resection and repair of the skull base defect with both muscle and bone graft and an artificial dural patch (Figure 5c). Histopathological examination of the tissue sample confirmed gliotic brain parenchyma and meningeal tissue herniation (Figure 6).

Herniated dura mater, brain parenchyma or both may be managed with resection or reduction into the intracranial cavity. Some authors claim that small viable hernias in a non-infected environment may be placed back inside the cranium. On the other hand, herniated brain matter is considered functionless, a result of long-standing ischaemia. For this reason, most authors agree that transection or resection of the pedicle, because encephalocoeles seldom contain functional brain tissue, and the sphenoidal portion is considered to be contaminated.^17,19,20^ It also has the benefit of removing the mucosa adherent to the wall of the sac, preventing mucocoele formation cephalad to the site of reconstruction.^21^ This tissue is also potentially epileptogenic.^22^

Our patient never presented with clinical signs or symptoms of cranial hypertension before or after the correction of the CSF leak. In fact, prior to surgery symptoms suggested intracranial hypotension interpreted as a consequence of CSF leakage (mild headache worsening with flexion posture of the head), which was completely solved after surgery. Also, there was no worsening on imaging suggesting hypertension (although those findings are thought to appear slowly over time). It is not clear which patients with evidence of elevated intracranial pressure may undergo isolated repair and which require the use of CSF diversion techniques, such as lumbar drainage, ventriculoperitoneal shunting and medical treatment;^18^ as the patient is asymptomatic and with no apparent consequences of intracranial hypertension, namely, papilloedema or visual disturbances, we believe there is no indication for any further medical or surgical intervention.

## Learning points

1. CSF spontaneous fistula should prompt investigation in order to exclude meningoencephaloceles.
2. The true transsphenoidal type is rarely reported, particularly through the Sternberg’s canal.
3. The clinical picture more often includes persistent CSF rhinorrhoea and recurrent meningitis. Obese middle-aged women seem to be more commonly affected and the suggested underlying mechanism hypothesizes benign intracranial hypertension to be a relevant factor.
4. MRI seems to be the most valuable imaging modality for an accurate diagnosis, although both CT and MRI are complementary modalities, especially for surgical planning.

## Consent

We, the authors, confirm that a patient’s relative has signed an informed consent regarding the submission of the CT, MRI and pathology images without identification.
